# Microalgae Oil Attenuates Liver Fat Deposition in NAFLD via Modulation of Anti‐Lipogenic Genes and Insulin Signaling Pathways in HFD Mice

**DOI:** 10.1002/fsn3.71882

**Published:** 2026-06-14

**Authors:** Athba AlQahtani, Mingjie Wang, Liping Liu, Liyuan Ran, Chao Liu, Yun Liang, Jinhui Yu, Yingjie Wu, Qingbo Guan

**Affiliations:** ^1^ Department of Endocrinology Shandong University Affiliated Shandong Provincial Hospital Jinan Shandong China; ^2^ Key Laboratory of Endocrine Glucose & Lipids Metabolism and Brain Aging, Ministry of Education Shandong Provincial Hospital, Science and Technology Innovation Centre, School of Laboratory Animal & Shandong Laboratory Animal Centre, Shandong First Medical University & Shandong Academy of Medical Sciences Jinan Shandong China; ^3^ Department of Endocrinology Affiliated Hospital of Inner Mongolia Medical University Hohhot China; ^4^ Gaoyou Integrated Traditional Chinese and Western Medicine Hospital Gaoyou China; ^5^ Institute of Genome Engineered Animal Models for Human Diseases, National Center of Genetically Engineered Animal Models for International Research Dalian Medical University Dalian Liaoning China; ^6^ Institute of Crop Germplasm Resources Shandong Academy of Agricultural Sciences Jinan China; ^7^ Plant Health Research Institute Qingdao China; ^8^ Department of Endocrinology and Nephrology PKUCare CNOOC Hospital Tianjin China

**Keywords:** cholesterol, dyslipidaemia, microalgae oil, NAFLD, obesity

## Abstract

Non‐alcoholic fatty liver disease (NAFLD) is a leading cause of liver‐related mortality and morbidity globally, with its prevalence steadily rising. DHA‐rich fish oil has demonstrated potential in managing NAFLD. However, little is known about the effect of pure DHA extracted from microalgae oil. This study explored the influence DHA‐rich microalgae oil might have on genes and metabolites associated with lipid and glucose metabolism in NAFLD mice, offering promising insights into a novel, cost‐effective treatment for NAFLD. In this study, C57BL/6J mice (*n* = 15) were used. NAFLD mice model was established by feeding mice HFD. Microalgae oil (10 μL/g body weight) was administered daily by oral gavage to HFD‐induced NAFLD mice (*n* = 5), while control mice and untreated HFD mice (*n* = 5 per group) received an equivalent volume of saline. Body weight was monitored regularly, and blood samples were collected to measure serum glucose and lipid levels. Tissue samples were analyzed using H&E staining and Oil Red O staining to assess histological changes. Differentially expressed genes and metabolites were identified through enrichment analyses. Microalgae oil supplementation showed a potential effect in reducing hepatic fat accumulation in NAFLD mice. The increase in liver weight induced by HFD was partially reduced by 7% in microalgae oil–treated mice. Moreover, microalgae oil partially restored 10% of the HFD‐induced loss in muscle mass and decreased white adipose tissue depots by an average of 39.3%. Treatment significantly lowered serum total cholesterol (from 4.39 to 3.57 mmol/L) and LDL‐c (from 0.98 to 0.63 mmol/L), while ameliorating hepatic enlargement and the elevation of liver enzymes (AST, ALT). RNA sequencing revealed that 79.8% of HFD‐upregulated genes were potentially downregulated by microalgae oil, particularly those related to glucose and lipid metabolism. Furthermore, microalgae oil treatment was associated with a downregulation of the PPAR and PI3K‐Akt signaling pathways, which were found to be enriched in the HFD‐fed group. Upregulation of *Irs1* and suppression of *Saa2* might suggest enhanced glucose uptake and reduced inflammation. Metabolomic analyses, including MSEA and OPLS‐DA, suggested that microalgae oil intervention may modulate the abundance of various carbohydrates and fatty acids. Collectively, these findings indicate that DHA‐enriched microalgae oil might aid in alleviating HFD‐induced metabolic and inflammatory disturbances in NAFLD. In conclusion, DHA‐enriched microalgae oil might exert a positive regulatory effect on genes and metabolites governing lipid and glucose metabolism. The findings from this study may provide a foundation for the development of a beneficial and cost‐effective microalgae oil as a potential therapeutic strategy. However, although the HFD‐induced NAFLD model recapitulates several features of human disease, it does not fully capture the complexity and heterogeneity of NAFLD in humans. The dosing regimen, involving a single dose administered via oral gavage, may not directly translate to typical human intake patterns, where intake occurs through diet and may influence absorption and metabolic responses. While beneficial effects were observed, the underlying molecular mechanisms require further investigation.

## Graphical abstract

Dietary microalgae oil might reverse hepatic lipid deposition in a mouse model of fatty liver. This intervention significantly improves the overall fatty liver condition by reducing lipid accumulation and simultaneously restoring insulin sensitivity.

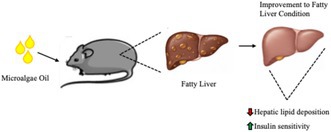

**Abbreviations**
ALT: alanine aminotransferase
AST: aspartate aminotransferase
DHA: docosahexaenoic acid
GO: Gene Ontology
KEGG: Kyoto Encyclopaedia of Genes and Genomes
LDL‐c: low density lipoprotein cholesterol
MSEA: Metabolite Set Enrichment
NAFLD: non‐alcoholic fatty liver disease
TBA: total bile acid
TC: total cholesterol
TG: triglycerides

## 1 Introduction

Non‐alcoholic fatty liver disease (NAFLD), also known as metabolic dysfunction‐associated steatotic liver disease (MASLD) in human pathology, is commonly associated with obesity. It is increasingly prevalent worldwide and is the leading cause of mortality and morbidity among liver diseases. Affecting about 38% of the global population, its prevalence is higher in men (40%) than women (26%) (Riazi et al. 2022). NAFLD involves fat accumulation in the liver, which is often harmless in its early stages but can progress to severe complications such as cirrhosis if left undetected. It is often linked to various health issues, including diabetes, hypertension, kidney diseases, and an increased risk of developing cardiovascular conditions. The etiology of NAFLD is multifactorial, involving obesity, insulin resistance, diabetes, inflammation‐induced oxidative stress, and unhealthy lifestyle behaviors (Frenette et al. 2021). High intake of refined carbohydrates, including starch, sucrose, and fructose commonly present in processed foods, in combination with dietary fats such as saturated fatty acids (e.g., palmitic acid, C16:0), monounsaturated fatty acids (e.g., oleic acid, C18:1*n* − 9), and *n* − 6 polyunsaturated fatty acids (e.g., linoleic acid, C18:2*n* − 6), plays a pivotal role in promoting hepatocellular lipid accumulation and the development of hepatic steatosis (Aune et al. 2023; Cao et al. 2024). Alcohol consumption can further increase carbohydrates and triglycerides to be delivered to the liver, leading to net lipid storage in the form of hepatic triglycerides. Dietary studies indicated that carbohydrates and fats are often consumed together across meals and snacks, resulting in rapid postprandial hyperglycaemia and increased insulin secretion, which stimulates hepatic de novo lipogenesis (Valenzuela et al. 2026). Lipotoxicity from visceral adipose tissue may also play a role in liver damage (Frenette et al. 2021). In addition, genetic factors such as variants in PNPLA3 (patatin‐like phospholipase domain‐containing 3) and TM6SF2 (transmembrane 6 superfamily member 2) significantly impact lipid metabolism. Other metabolic genes including FAS (fatty acid synthase) and PPARα (peroxisome proliferator‐activated receptor α) contribute to NAFLD pathogenesis (Jegatheesan et al. 2015). Given the strong link between obesity and NAFLD, weight reduction has been shown to significantly improve management, with or without dietary and anti‐diabetic interventions (Kosmalski et al. 2023). Medications such as dapagliflozin and pioglitazone have also demonstrated effectiveness in treating NAFLD (Yoneda et al. 2021; Phrueksotsai et al. 2021). Dietary supplements, known as nutraceuticals, can provide essential micronutrients to individuals with insufficient dietary intake. Omega‐3 polyunsaturated fatty acids (PUFAs), a key nutraceutical, have shown promising effects in managing NAFLD and other liver conditions (Cussons et al. 2009; Manousopoulou et al. 2019; Scorletti et al. 2014). Sufficient levels of omega‐3 PUFAs have shown to reduce triglycerides and low‐density lipoprotein (LDL) synthesis in the liver, lowing hypertriglyceridemia risk. Furthermore, omega‐3 PUFAs enhance insulin sensitivity and inhibit hyperglycaemia by modulating PPARα activity (Jang and Park 2020). Nutritional interventions have demonstrated significant benefits in fatty liver disease management, including reductions in serum transaminases and intrahepatic lipid accumulation (Valenzuela et al. 2026). A meta‐analysis of 22 randomized controlled trials (RCTs) reported that omega‐3 PUFA supplementation significantly improved multiple metabolic outcomes in individuals with MASLD, including decreases in triglycerides, total cholesterol, and body mass index (BMI), alongside increases in high‐density lipoprotein (HDL) levels (Lee and Park 2024). Consistently, a separate meta‐analysis of seven RCTs involving 439 participants demonstrated that fish oil supplementation led to improvements in triglyceride levels, aspartate aminotransferase (AST), insulin resistance as assessed by HOMA‐IR, and waist circumference (Zhou et al. 2025). Collectively, these findings support omega‐3 PUFAs as a promising therapeutic approach for alleviating hepatic steatosis and associated metabolic disturbances.

Microalgae oil is a sustainable, plant‐based source of omega‐3 PUFAs, particularly eicosapentaenoic acid (EPA, C20:5(*n* − 3)) and docosahexaenoic acid (DHA, C22:6(*n* − 3)). It may offer advantages over animal‐derived oils due to its high omega‐3 yield and suitability for vegetarians. Industrially, microalgae exhibit rapid biomass growth, can be cultivated on non‐agricultural lands or in saline water, and accumulate lipids under stress conditions while continuing to produce valuable proteins and pigments (Choudhary et al. 2017). These characteristics make microalgae oil an efficient and scalable source of DHA‐rich biomass. DHA (C22:6(*n* − 3)) is recognized for its beneficial effect on regulating liver lipid metabolism and alleviating NAFLD. Previous studies, predominantly utilizing fish oil, have reported that DHA supplementation reduced liver fat and improved hepatic fibrosis (Valenzuela and Videla 2020; Kelley 2016). Moreover, apolipoprotein E‐deficient mice exhibited a reduction in serum lipid profiles and liver deposition following treatment with DHA‐containing fish oil (Xu et al. 2021). However, studies specifically investigating DHA‐rich microalgae oil are limited. We have previously reported that purified microalgae oil had a pure high DHA content (Ran et al. 2022). As microalgae oil provides a sustainable and industrially efficient source of DHA, offering content comparable to traditional salmon‐based supplements (Arterburn et al. 2008), this study evaluates its therapeutic impact on NAFLD. We further investigate how DHA‐enriched microalgae oil modulates key genes and metabolites involved in lipid and glucose metabolism. These findings may support the development of effective, scalable treatments for NAFLD.

## 2 Materials and Methods

### 2.1 Ethics Statement

All animal experiments were conducted in accordance with the National Institutes of Health (NIH) Guidelines on the Use of Laboratory Animals and approved by the Experimental Animals Committee of Shandong Provincial Hospital (ethical approval no. 2022‐007).

### 2.2 Experimental Animals

Male C57BL/6J mice (6 weeks old) were purchased from Beijing Vital River Laboratory Animal Technology Co. and housed in a controlled environment with a 12 h light/dark cycle, free access to food and water, and no more than 5 mice per cage. Control mice were fed a regular chow diet (Keao Xieli Feed Ltd., China) with 70% carbohydrates, 20% protein and 10% fat (kcal). The NAFLD mouse model was established by feeding mice a high‐fat diet (HFD, D12492 Research Diets Inc., USA) with 20% carbohydrates, 20% protein, and 60% fat (kcal) for 8 weeks. No additional dietary modifications were made during the intervention. Food intake was not quantitatively recorded in this study. Although no overt differences in feeding behavior were visually observed, its potential influence on metabolic outcomes cannot be excluded. Microalgae oil (10 μL/g body weight) was administered daily by oral gavage to HFD‐induced NAFLD mice (*n* = 5), while control mice and untreated HFD mice (*n* = 5 per group) received an equivalent volume of saline. Body weight was monitored every day, and NAFLD was confirmed via blood tests and liver morphology analysis. Blood samples were collected for measurements of glucose, total cholesterol (TC), triglycerides (TG), low density lipoprotein cholesterol (LDL‐c), aspartate aminotransferase (AST), alanine aminotransferase (ALT), and total bile acid (TBA) according to the manufacturer's instructions using an automatic biochemical analyzer (A110‐1 & A111‐1 Jian Cheng Biological Engineering Institute, Nanjing, China). Blood collection and testing was explained in more detail in a previous publication (Yu, Ma, et al. 2017).

### 2.3 Microalgae Oil

Microalgae oil extracted from *Schizochytrium* sp. was obtained from Tiankai Biological Technology Co. Ltd. (No. 3200/16040, Jiangsu, China) and subsequently purified by medium‐pressure flash chromatography using a high‐voltage glass C18 column (70 × 460 mm^2^; Biotage, USA). It was freshly prepared and purified for the current experiments. Purification was performed using water (A) and methanol (B) as the mobile phase, with 90% B from 0 to 160 min followed by 100% B at 160 min, at a flow rate of 100 mL/min; the sample load was 25 mL in 10 mL tetrahydrofuran, and UV detection was set at 214 nm as previously reported (Yu, Wang, et al. 2017). The optimized extraction process for *Schizochytrium* algal oil was carbon dioxide flow rate 30 L/h, separation pressure 5 MPa, separation temperature 35°C, co‐solvent ratio 1:1, extraction pressure 35 MPa, extraction temperature 40°C, and extraction time 2 h. The complete fatty acid profile of microalgae oil has been reported previously (Yu, Wang, et al. 2017). Briefly, DHA (C22:6(*n* − 3)) was dominant in microalgae oil (97.8%), and the proportion of EPA (C20:5(*n* − 3)) as well as saturated fatty acids (SFAs) and monounsaturated fatty acids (MUFAs) was as low as 1%.

### 2.4 Metabolic Chamber

Oxygen consumption and carbon dioxide production were measured using a metabolic chamber equipped with a voluntary running wheel. Gas exchange was recorded during each stage of the exercise. Mice were acclimatized in the chambers for at least 24 h prior to measurement to minimize stress. Each mouse was placed in an individual metabolic chamber with adequate ventilation to prevent CO_2_ accumulation. Respiratory Exchange Ratio (RER) was calculated with values near 0.7 indicating fat oxidation and values near 1.0 indicating carbohydrate oxidation.

### 2.5 Glucose Tolerance and Insulin Tolerance Tests (GTT and ITT)

GGT and ITT were performed as previously described (Yu, Ma, et al. 2017). Glucose (2 g/kg; Sigma Aldrich, Saint Louis, MO) was intraperitoneally administered for GGT, and insulin (0.75 U/kg; Humulin R, Eli Lilly, Indianapolis, IN) for ITT. Blood glucose levels were monitored at 15, 30, 60, 90, 120 min post‐injection using an automated glucometer (Roche, Basel, Switzerland).

### 2.6 Oil Red O Staining

Frozen tissue sections were fixed with 4% PFA for 30 min then immersed in 60% isopropanol and rinsed with distilled water. The sections were then stained with a 3:2 ratio Oil Red O working solution for 10 min, protected from light. Nuclei were stained with hematoxylin for 1–2 min. Tissue sections were mounted with warm glycerol gelatine and examined under the microscope.

### 2.7 Hematoxylin and Eosin (H&E) Staining

Liver tissue sections (5 μm) were fixed in 10% neutral buffered formalin. The embedded sections were immersed in hematoxylin solution for 5–10 min then rinsed. The sections were stained with eosin solution for 1–2 min and rinsed with distilled water. Tissue morphology was examined under a light microscope (Nikon, Tokyo, Japan), with three tissue fields randomly selected and examined from each mouse.

### 2.8 Analysis of Differentially Expressed Genes and Metabolites

Differentially expressed genes and metabolites between the groups (control, HFD and HFD treated with microalgae oil) were analyzed using Gene Ontology (GO), Kyoto Encyclopaedia of Genes and Genomes (KEGG), and Metabolite Set Enrichment Analyses (MSEA). For metabolite extraction, a 10 mg liver tissue sample was mixed with methanol containing an internal standard. The supernatant from the homogenized samples was transferred to a 96‐well plate, with an internal standard added to each well. Derivative standards were serially diluted and added to the wells prior to sealing the plate ready for LC–MS analysis.

### 2.9 Statistical Analysis

The data were presented as mean ± SEM and analyzed using analysis of variance (ANOVA) for multiple comparisons. Statistical significance was defined as *p < 0.05, **p < 0.01 and ***p < 0.001. Enrichment analysis and supervised orthogonal partial least squares discriminant analysis (OPLS‐DA) were performed using R (version 4.4.1). Metabolite Set Enrichment Analysis (MSEA) analysis was used to investigate the differential abundance of liver metabolites.

## 3 Results

### 3.1 DHA‐Rich Microalgae Oil Might Aid in Improving Glucose Tolerance and Insulin Sensitivity in NAFLD Mice

**FIGURE 1:**
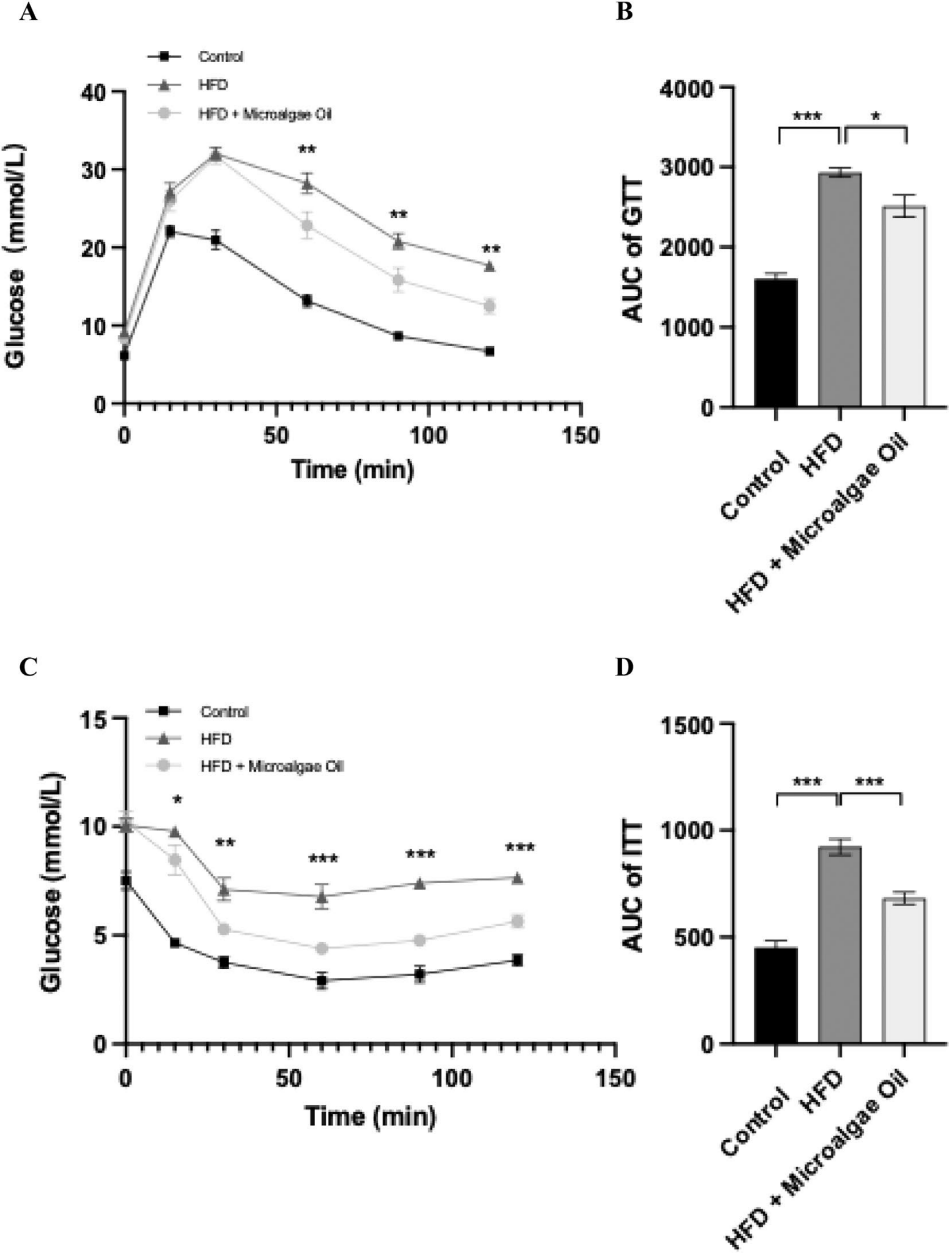
DHA‐rich microalgae oil improves glucose tolerance and insulin sensitivity. (A) Glucose tolerance tests (GTT) measured in mice after a 2 g/kg glucose load. Basal blood glucose was determined after 12 h of fasting. Then glucose level was measured at various intervals: 15, 30, 60, 90, 120 min. (B) The graph represents area under the curve (AUC) of GTT in the control, HFD, and microalgae oil‐treated groups. (C) Insulin tolerance test (ITT) was determined after 8 h of fasting prior to a 0.75 U/kg insulin injection. Blood glucose levels were detected at various intervals: 15, 30, 60, 90, 120 min. (D) The graph represents area under the curve (AUC) of ITT in the control, HFD, and microalgae oil‐treated groups. Data represent mean ± SEM; ANOVA significance was considered at *p < 0.05, **p < 0.01 and ***p < 0.001.

To assess the impact of DHA‐rich microalgae oil on glucose metabolism, GTT was performed to measure changes in glucose levels over time after a glucose load. As shown in Figure 1A,B, the control group cleared glucose from the bloodstream more efficiently than the HFD group where glucose tolerance was impaired, as indicated by elevated glucose levels over time, suggesting reduced capacity for glucose clearance. Notably, there was a significant improvement to glucose clearance in the microalgae oil‐treated group. Impaired glucose metabolism in the HFD group may indicate onset of metabolic disorders like insulin resistance or type 2 diabetes. To further analyze this, ITT was conducted (Figure 1C,D). As anticipated, the HFD group showed significantly higher ITT values, indicating reduced insulin sensitivity. However, microalgae oil treatment showed a significant improvement in insulin sensitivity. These data suggest that microalgae oil may help improve glucose tolerance and insulin sensitivity in NAFLD mice.

### 3.2 Microalgae Oil Mitigates Hepatic Fat Deposition in NAFLD Mice

**FIGURE 2:**
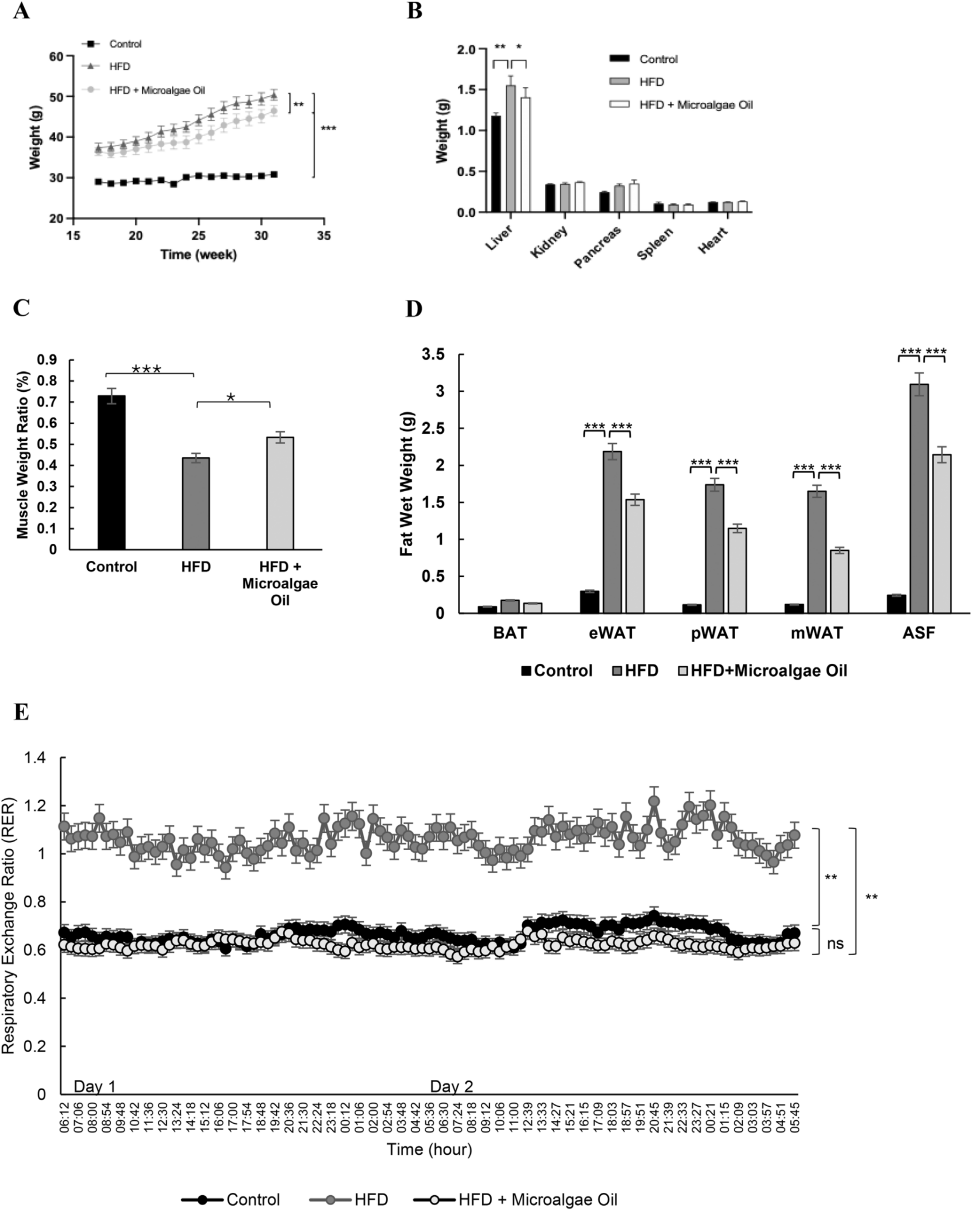
The protective effects of microalgae oil in NAFLD. (A) Changes in measurements of body weight in response to different diets. Male C57BL/6J mice (6 weeks old) were used (total *n* = 15). Control mice were fed regular chow diet (*n* = 5) whereas NAFLD mice model was established by feeding mice high‐fat diet (*n* = 10). 10 μL/g of microalgae oil solution was administered to NAFLD mice for another 8 weeks on a daily basis (*n* = 5). The control and HFD groups were given 10 μL/g of saline. (B) Measurement of various organs (liver, heart, pancreas, spleen, and kidney) to detect changes in weight in response to microalgae oil treatment. (C) The ratio of muscle weight relative to body weight in control, HFD, and microalgae oil treated groups. (D) Measurement of fat wet weight in control, HFD, and HFD treated with microalgae oil. Key: BAT, brown adipose tissue; eWAT, epididymal white adipose tissue; pWAT, perirenal white adipose tissue; mWAT, mesenteric white adipose tissue; ASF, abdominal subcutaneous fat. (E) The respiratory exchange ratio (RER) over time between control, HFD, and HFD treated with microalgae oil groups. RER was reflected by the ratio of carbon dioxide produced (vCO_2_) and oxygen consumed (vO_2_) throughout day and night. Data represent mean ± SEM; ANOVA significance was considered at *p < 0.05, **p < 0.01, and ***p < 0.001; ns denotes non‐significant.

NAFLD‐induced mice by HFD experienced a significant weight reduction following treatment with microalgae oil compared to the untreated HFD group (Figure 2A). Various organs including liver, heart, pancreas, spleen, and kidney were measured to detect any weight changes in response to microalgae oil treatment. As shown in Figure 2B, no significant changes were observed in the weight of the heart, spleen, pancreas, or kidneys. However, the significant increase in liver weight observed in the HFD group was partially augmented by 7% in the microalgae oil treated group. Additionally, microalgae oil supplementation mitigated the reduction in muscle mass caused by HFD and restored approximately 10% of muscle mass (Figure 2C). Although microalgae oil had no major effect on BAT mass, it significantly caused a reduction in other white adipose tissue depots by an average of 39.3%; specifically, a 29.8% reduction in eWAT, 34.2% in pWAT, 48.7% in mWAT, and 44.4% ASF (Figure 2D). The predominant macronutrients (carbohydrates or fats) being metabolized for energy were assessed using RER (Figure 2E). The RER fluctuated closer to 1.0 in the HFD, indicating a higher reliance on carbohydrate metabolism. Microalgae oil supplementation seemed to lower RER values (approx. 0.7), indicating a predominant shift toward lipid metabolism.

**FIGURE 3:**
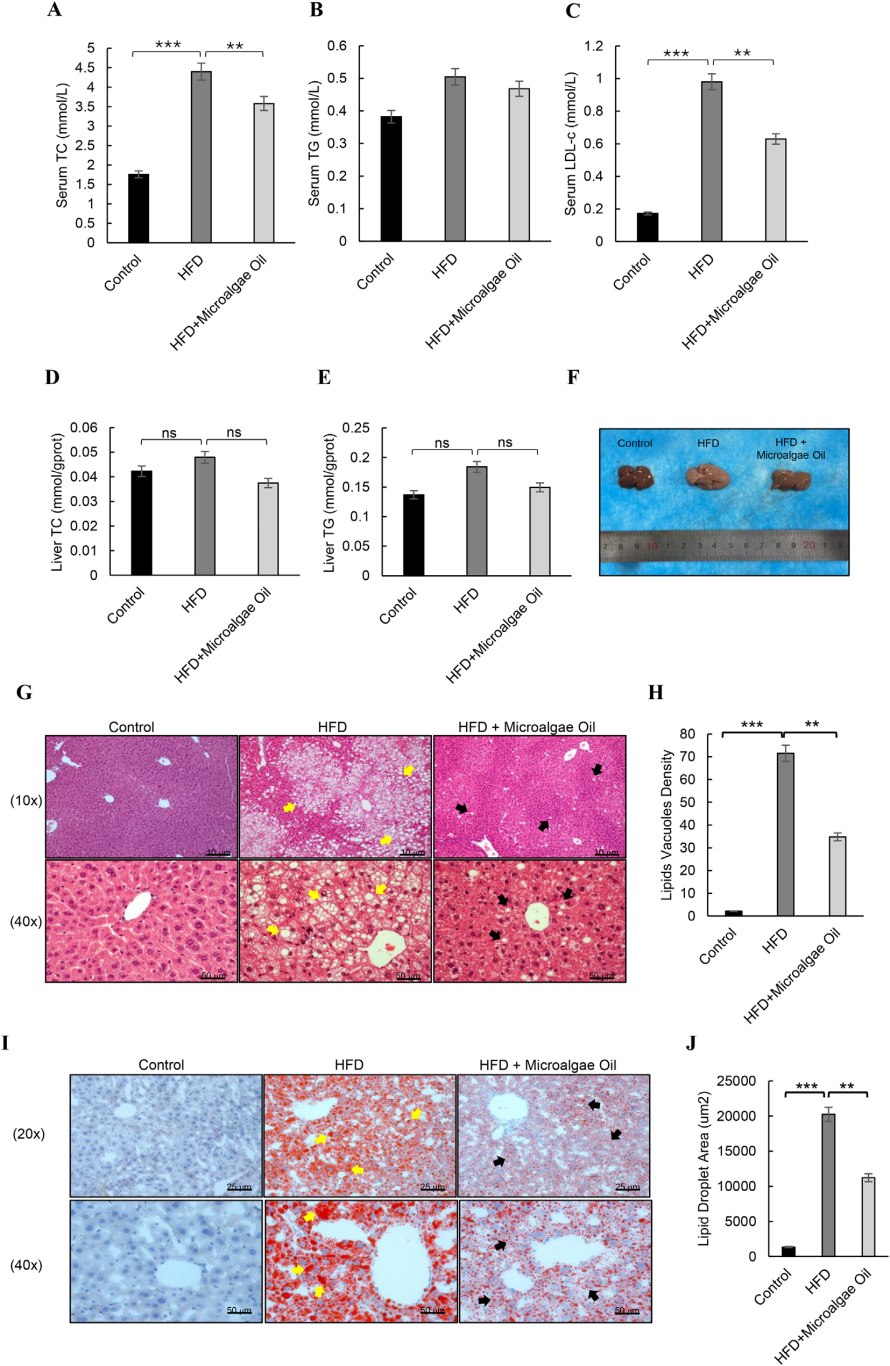
The protective effect of microalgae oil on serum lipids and liver fat deposition. Measurements of total cholesterol (TC) (A), triglycerides (TG) (B), and low‐density lipoprotein cholesterol (LDL‐c) (C) levels in blood serum samples. (D, E) Liver total cholesterol (TC) and triglycerides (TG) measured using ELISA kits in the three groups: Control, HFD, and HFD supplemented with microalgae oil. (F) The morphology of liver tissue in control, HFD, and HFD treated with microalgae oil. (G) H&E staining of liver tissue conducted to examine the extent of steatosis; imaged at 10× and 40× in control, HFD, HFD supplemented with microalgae oil. Black arrows denote microvesicular steatosis, while yellow arrows denote macrovesicular steatosis. (H) Lipid vacuoles density in the presence and absence of microalgae oil. Liver fat deposition was represented by various considerable vacuoles. (I) Oil red O staining visualized at 20× and 40× using bright microscope. Black arrows denote microvesicular steatosis, while yellow arrows denote macrovesicular steatosis. (J) Lipid droplet areas were determined in control, HFD, and HFD treated with microalgae oil groups. Data represent mean ± SEM; ANOVA significance was considered at **p < 0.01, and ***p < 0.001, non‐significance indicated as (ns).

Microalgae oil significantly improved key serum lipid markers, reducing those elevated by the HFD. Total cholesterol was substantially higher in the HFD group (4.39 mmol/L) than controls (1.75 mmol/L), but microalgae oil intervention significantly decreased it to (3.57 mmol/L) (Figure 3A). Similarly, the HFD‐induced increase in LDL‐c (0.98 mmol/L) was significantly lowered by microalgae oil to (0.63 mmol/L) (Figure 3C). While serum triglycerides were slightly higher in the HFD and treated groups compared to controls (0.38 mmol/L), the differences were not statistically significant (Figure 3B). Overall, the data confirm the efficacy of microalgae oil in mitigating HFD‐induced increases in serum total cholesterol and LDL‐c in NAFLD mice. Hepatic lipid levels and liver morphology were assessed to complement the serum data. Liver cholesterol levels were highest in the HFD group (0.048 mmol/gprot), followed by controls (0.042 mmol/gprot), and were lowest in the microalgae oil group (0.037 mmol/gprot) (Figure 3D); however, these differences were not statistically significant. Similarly, liver triglycerides were highest in the HFD group (0.184 mmol/gprot), but the microalgae oil group (0.149 mmol/gprot) showed levels closer to the control baseline (0.136 mmol/gprot) (Figure 3E). Morphological analysis revealed that HFD‐fed mice exhibited marked pale hepatomegaly (3.0 cm in width), indicative of liver steatosis and damage. Conversely, the microalgae oil‐treated group showed an intermediate size (2.0 cm in width) and darker color, suggesting a moderate protective effect on liver morphology by preventing further enlargement, though not fully reversing the HFD‐induced damage (Figure 3F). Histological analysis further supported the protective effect of microalgae oil on the liver. H&E staining revealed severe steatosis in the HFD group, characterized by a significant increase in large lipid vacuoles and fat deposition, whereas the control tissue was normal (Figure 3G,H). The microalgae oil‐treated group still showed lipid accumulation, but the vacuoles were observably smaller and less frequent than those in the HFD group, suggesting a reduction in the severity of lipid accumulation. This was confirmed by Oil Red O staining, which showed moderate lipid accumulation in the microalgae oil group, markedly less extensive than the significant accumulation seen in the HFD group, and minimal accumulation in controls (Figure 3I,J). Collectively, these findings support that microalgae oil has a protective effect in mitigating hepatic fat deposition and liver damage in NAFLD mice.

### 3.3 Microalgae Oil Augments HFD‐Induced Increase in Liver Enzymes

**FIGURE 4:**
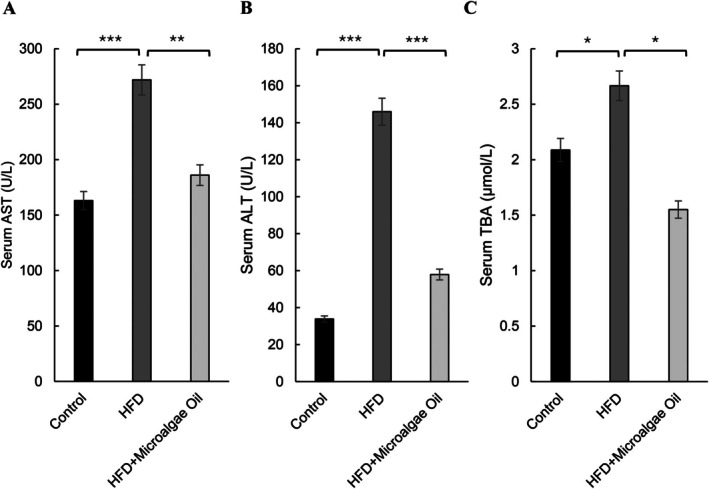
The effect of microalgae oil on liver function. Blood samples were collected from mice grouped as control, HFD, and HFD supplemented with microalgae oil group. Levels of (A) aspartate aminotransferase (AST), (B) alanine aminotransferase (ALT), and (C) total bile acid (TBA) levels in serum were determined. Data represent mean ± SEM; ANOVA significance was considered at *p < 0.05, **p < 0.01 and ***p < 0.001.

Liver enzymes aspartate transaminase (AST) and alanine transaminase (ALT) were significantly elevated in the HFD group (271.9 U/L) and (145.97 U/L), respectively, compared to controls (Figure 4A,B). Microalgae oil treatment effectively reduced AST levels to 186.0 U/L (i.e., a reduction of 31.6%) and ALT levels to 57.33 U/L (i.e., 60.7% reduction) compared to the levels in HFD group, indicating improved liver function. Similarly, high serum Total Bile Acids (TBA) in the HFD group (2.66 μmol/L), reflective of dysregulated metabolism, were significantly lowered by microalgae oil treatment to 1.55 μmol/L, comparable to the control level of 2.08 μmol/L (Figure 4C). These data confirm the efficacy of microalgae oil in restoring key indicators of liver health and bile acid metabolism in NAFLD mice.

### 3.4 Microalgae Oil Reverses HFD‐Induced Transcriptional Changes and Modulates Glucose‐ and Lipid‐Related Gene Expression

**FIGURE 5:**
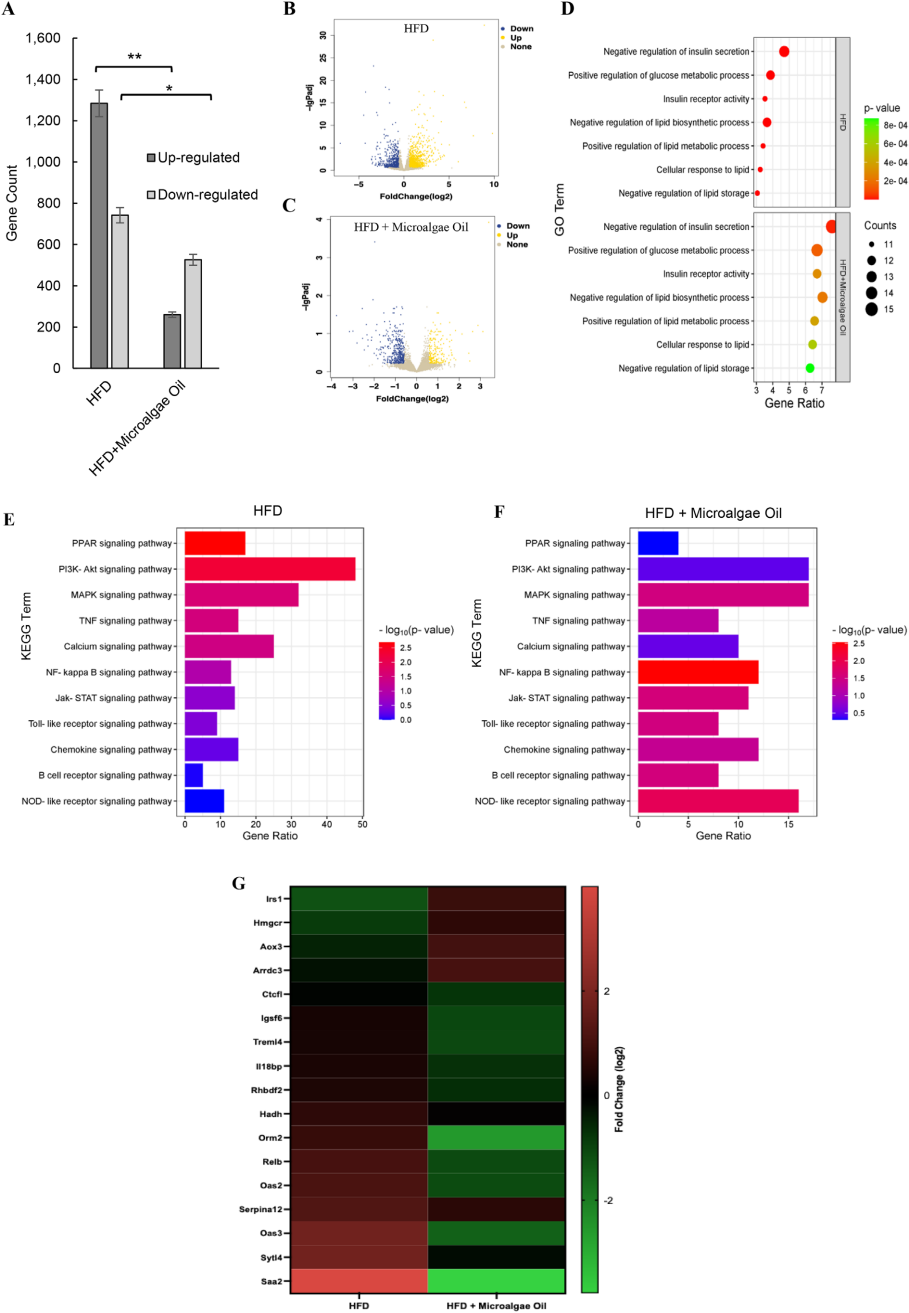
Modulation of glucose and lipid metabolic gene profiling in response to microalgae oil treatment. Illustration of the total gene counts (A) and log_2_ fold change (B, C) of up‐regulated and down‐regulated genes in response to HFD with and without microalgae oil supplementation. (D) Gene ontology (GO) analysis showing changes in expression of genes involved in glucose and lipid metabolism. (E) KEGG analysis of various signaling pathways in HFD group relative to the control group. (F) KEGG analysis of signaling pathways after treatment with microalgae oil. The graph is relative to the HFD group. (G) Profiling of genes ranked by level of expression in log_2_ fold change between the HFD group and HFD treated with microalgae oil.

RNA sequencing revealed a pronounced regulatory effect of microalgae oil on gene expression alterations induced by a high‐fat diet. Transitioning from a regular diet to HFD resulted in the differential expression of more than 2000 genes, including 1284 upregulated and 742 downregulated transcripts. Notably, microalgae oil supplementation reversed a substantial proportion of these changes, with 79.8% of genes that were initially upregulated by HFD subsequently downregulated or suppressed (Figure 5A–C). GO enrichment analysis showed that this regulation may target glucose and lipid metabolic processes. Microalgae oil treatment specifically activated genes involved in insulin secretion and lipid biosynthetic pathways and led to an observed increase in insulin receptor activity, suggesting improved insulin sensitivity. Furthermore, genes associated with lipid storage, which were upregulated in the HFD group, were notably downregulated by microalgae oil (Figure 5D). KEGG enrichment analysis, complementing GO findings, demonstrated that microalgae oil significantly modulated signaling pathways central to metabolic and inflammatory homeostasis (Figure 5E,F). The HFD enriched the PPAR signaling pathway, suggesting lipid dysregulation, as well as the PI3K‐Akt signaling pathway. In contrast, microalgae oil treatment downregulated the enrichment of both PPAR and PI3K‐Akt signaling pathways. Gene expression analysis further confirmed these regulatory effects (Figure 5G). For instance, *Irs1* gene expression was increased in response to microalgae oil treatment, which might suggest improved glucose uptake and enhanced insulin sensitivity. *Saa2* was highly expressed in the HFD group but was reduced by microalgae oil treatment, indicating a potential role of microalgae oil in mitigating inflammation or stress responses induced by the high‐fat diet. *Arrdc3, Hadha, and Serpina12* were downregulated in response to a high‐fat diet and remained unaffected by microalgae oil supplementation. These genes are known to be involved in lipid metabolism (*Arrdc3*), fatty acid oxidation (*Hadha*), and insulin sensitivity (*Serpina12*). Together, these findings suggest that microalgae oil might be protective against NAFLD possibly by mediating metabolic pathways pertaining to glucose and lipid homeostasis.

### 3.5 Microalgae Oil Might Modulate Liver Metabolites

**FIGURE 6:**
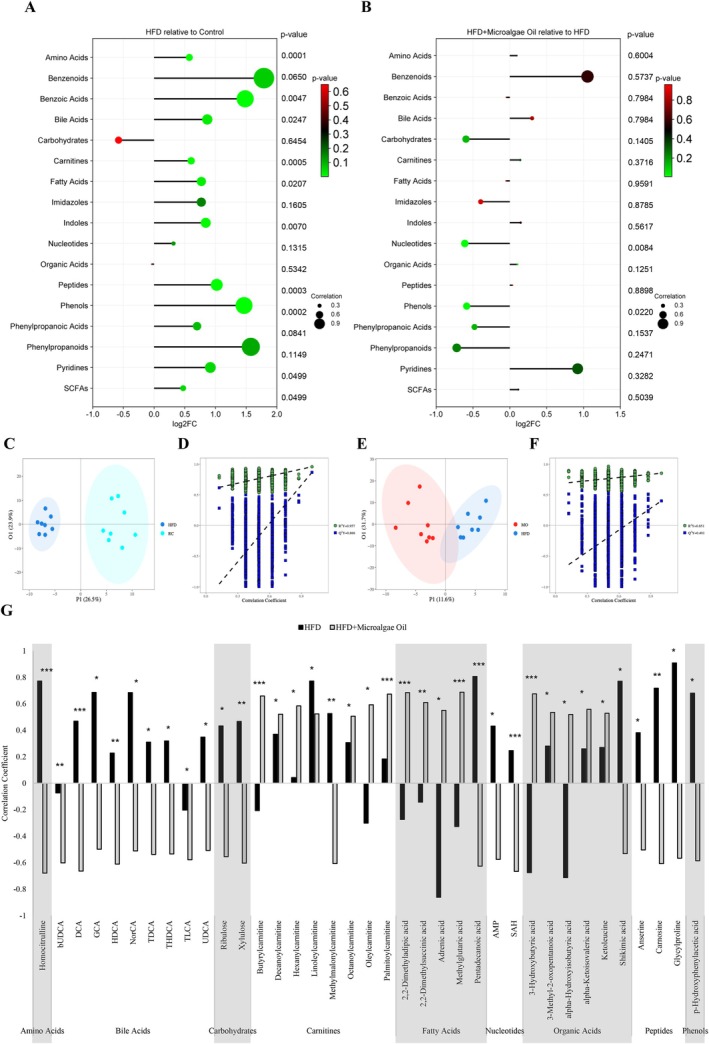
The effect of microalgae oil on liver metabolites. The proportional contribution of each metabolite class to the total metabolite pool in the HFD group relative to control group (A) and microalgae oil‐ treated group relative to HFD group (B). MSEA analysis was used, and data were expressed as log_2_ fold changes (log_2_FC) for different metabolite classes, with *p*‐values represented next to each class. Key: SCFAs, short‐chained fatty acids. (C) OPLS‐DA of liver metabolites clusters of two groups: Control and HFD. Ellipses represent 95% confidence regions. Control group identified as RC in light blue, HFD in dark blue. (D) Validation of OPLS‐DA model using permutation testing for control and HFD groups. The original model achieved an *R* ^2^ *Y* value of 0.957 and a *Q* ^2^ *Y* value of 0.866. (E) OPLS‐DA of liver metabolite clusters of two groups: HFD and HFD treated with microalgae oil. Ellipses represent 95% confidence regions. HFD group identified in dark blue, HFD treated with microalgae oil (MO) in red. (F) Validation of the OPLS‐DA model using permutation testing for HFD and microalgae oil‐treated groups. The original model achieved an *R* ^2^ *Y* value of 0.851 and a *Q* ^2^ *Y* value of 0.401. (G) Correlation analysis between liver metabolites under the presence and absence of microalgae oil treatment in HFD mice. Bars express correlation coefficient in HFD group (black) and microalgae oil treated group (gray). Statistical significance using ANOVA was identified at *p < 0.05, **p < 0.01, and ***p < 0.001.

MSEA and OPLS‐DA analyses were employed to determine the overall differential variance and identify key drivers of changes in liver metabolites. Alterations in liver metabolites were observed in the HFD group (Figure 6A,B) in which fatty acids (*p* = 0.0207) and bile acids (*p* = 0.0247) were significantly upregulated, while amino acids (*p* = 0.0001) were downregulated relative to the control group. Microalgae oil partially mitigated these HFD‐induced changes by increasing carbohydrate metabolism and reducing fatty acid abundance. OPLS‐DA analysis separated the HFD and control groups into distinct clusters (Figure 6C). This model demonstrated high robustness and excellent predictive ability (R^2^Y value of 0.957 and a Q^2^Y value of 0.866) (Figure 6D). When comparing the microalgae oil‐treated group to the HFD group, the samples clustered distinctly, though with some overlap (Figure 6E), which might suggest a partial but not complete reversal of the HFD‐induced metabolite profile. This second model was also confirmed to be robust (R^2^Y value of 0.851 and a Q^2^Y value of 0.401) (Figure 6F). Furthermore, correlation analysis revealed distinct patterns between the HFD group and the group supplemented with DHA‐rich microalgae oil within the fatty acid panel (Figure 6G). Under HFD conditions, 2,2‐dimethyladipic acid, 2,2‐dimethylsuccinic acid, adrenic acid, and methylglutaric acid all demonstrated negative correlation coefficients, with adrenic acid exhibiting the strongest negative association among the fatty acids analyzed. In contrast, pentadecanoic acid showed a strong positive correlation in the HFD group. In the microalgae oil‐supplemented group, the correlation pattern was reversed. Specifically, 2,2‐dimethyladipic acid, 2,2‐dimethylsuccinic acid, adrenic acid, and methylglutaric acid displayed positive correlation coefficients, whereas pentadecanoic acid showed a negative correlation. Among these metabolites, adrenic acid demonstrated the largest magnitude shift in correlation direction between the two groups. Overall, all five fatty acids exhibited a change in correlation direction when comparing HFD alone to HFD supplemented with microalgae oil. Supplementation with microalgae oil also revealed marked shifts in both direction and magnitude across multiple metabolite classes. Among amino acids, homocitrulline exhibited a strong positive correlation under HFD, which shifted to a moderate‐to‐strong negative correlation following supplementation, indicating a possible directional inversion. Bile acids showed a uniform and complete reversal: all measured bile acids, including bUDCA, DCA, GCA, HDCA, NorCA, TDCA, THDCA, TLCA, and UDCA, were positively correlated under HFD and became negatively correlated in the supplemented group. Similarly, the carbohydrates ribulose and xylulose shifted from moderate positive correlations under HFD to moderate negative correlations with supplementation, reflecting consistent directional inversion. Carnitines displayed a mixed response pattern. While Methylmalonylcarnitine and Oleylcarnitine inverted from positive or negative correlations to opposite directions, other carnitines, such as Decanoylcarnitine, Octanoylcarnitine, Linoleylcarnitine, and Palmitoylcarnitine, remained positively correlated, though with varying magnitudes. Butyrylcarnitine shifted from a negative correlation to positive with supplementation, highlighting the heterogeneous effects within this metabolite class. Nucleotides also displayed directional inversion, with AMP and SAH positively correlated under HFD and negatively correlated in the supplemented group. Organic acids presented a mixed pattern: most retained positive correlations across both conditions, but alpha‐Hydroxyisobutyric acid inverted from negative to positive, and Shikimic acid reversed from strong positive to negative. Peptides uniformly inverted direction: Anserine, Carnosine, and Glycylproline were positively correlated under HFD but negatively correlated following supplementation. The phenol p‐Hydroxyphenyllactic acid also shifted from moderate positive to negative correlation (Figure 6G). Overall, microalgae oil supplementation induced possible directional reversal in bile acids, fatty acids, peptides, nucleotides, carbohydrates, and phenols, while carnitines and organic acids displayed mixed responses. Several metabolites, including Adrenic acid, Shikimic acid, and Glycylproline, exhibited substantial shifts in correlation magnitude. Collectively, these findings might suggest that microalgae oil modulates key energy metabolic pathways, enhancing the efficiency of lipid and carbohydrate metabolism in NAFLD.

## 4 Discussion

The global rise in overweight and obesity is a significant public health challenge, closely linked to metabolic disorders such as insulin resistance, dyslipidaemia, and NAFLD. These metabolic disruptions, exacerbated by high‐fat diets and sedentary lifestyles, underscore the urgent need for innovative, safe, and effective therapeutic options. To date, there are no specific FDA‐approved drugs for treating MASLD (Wang et al. 2023). Lipid‐lowering medications like atorvastatin and fenofibrate are used in clinic but may cause adverse effects (Wang et al. 2023). While vitamin C, lifestyle, and dietary changes could aid in MASLD management, poor patient compliance remains a challenge. Hence, this study demonstrated the potential of DHA‐rich microalgae oil as a natural therapeutic agent in mitigating the negative metabolic consequences of MASLD. The insights could also contribute to the development of beneficial and cost‐effective MASLD treatments.

Clinical data analyses indicated that supplementing the diet with long‐chain omega‐3 polyunsaturated fatty acids (PUFAs) was beneficial for MASLD patients. A meta‐analysis of nine studies confirmed that omega‐3 supplementation effectively decreased liver fat (Parker et al. 2012), and several clinical trials supported its use for lowering liver fat in both children and adults with MASLD (Nobili et al. 2011; Scorletti et al. 2014). Out of 235 clinical trials focused on nonalcoholic steatohepatitis (NASH) therapies, 23 utilized omega‐3 PUFAs as a treatment. Most of these trials used either fish oil or a combination of EPA and DHA for supplementation, with few studies investigating EPA or DHA alone. We have previously reported that microalgae oil derived from *Schizochytrium* species, rich in PUFAs such as EPA and DHA, proved to be an effective alternative to fish oil in managing obesity (Yu, Ma, et al. 2017). We showed that omega‐3 PUFAs significantly reduced abdominal fat accumulation, improved lipid profiles, and alleviated inflammation in the adipose tissue in HFD mice (Yu, Ma, et al. 2017). In this study, we further showed that microalgae oil supplementation reduced body and liver weight while increasing muscle mass in HFD‐induced NAFLD mice. High‐fat diet promoted substantial fat accumulation in white adipose tissue depots (eWAT, pWAT, mWAT, and ASF), which were hallmarks of obesity. Microalgae oil supplementation aided in lowering fat accumulation in these depots and improved lipid metabolism. While HFD primarily enhanced carbohydrate metabolism, microalgae oil caused a metabolic shift redirecting the system toward lipid metabolism and exerted hepatoprotective effects by lowering liver enzymes ALT and AST. Previous studies support the hepatoprotective effects of omega‐3 fatty acids in HFD‐induced liver injury. For example, supplementation with long‐chain omega‐3 PUFAs combined with extra virgin olive oil for 12 weeks was shown to attenuate hepatic steatosis in HFD‐fed mice, accompanied by improvements in antioxidant capacity, reduced inflammatory signaling, enhanced insulin sensitivity, and restoration of hepatic lipid metabolic balance (Valenzuela et al. 2016). In hepatic steatosis, changes in both the production and levels of omega‐3 PUFAs are a key aspect of disease progression. Conditions like NAFLD are marked by disruptions in PUFA metabolism, including reduced omega‐3 PUFA content in the liver and alterations in the enzymes responsible for their biosynthesis. Studies indicated that maintaining a balanced omega‐6/omega‐3 PUFA ratio helped prevent NAFLD, while an imbalance in this ratio and lower omega‐3 PUFA levels were associated with the development of hepatic steatosis and impaired lipid metabolism. Specifically, in fatty liver, omega‐3 PUFA synthesis or availability is decreased, reflecting suppression of endogenous PUFA biosynthetic pathways and a reduced hepatic omega‐3/omega‐6 ratio. This reduction was linked to increased triglyceride accumulation and abnormal lipid profiles in hepatocytes, suggesting that diminished omega‐3 PUFA production contributed to the lipotoxic environment characteristic of steatotic liver (Videla et al. 2022). Overall, these alterations in PUFA metabolism highlight the metabolic disturbances in steatosis and emphasize the importance of omega‐3 PUFAs in regulating hepatic lipid balance.

Gene expression analyses demonstrated that both DHA and EPA reduced the liver's inflammatory response to a Western diet though DHA proved more effective (Depner, Philbrick, and Jump 2013; Depner, Traber, et al. 2013). These studies indicated that omega‐3 PUFAs, particularly DHA, function by downregulating key cellular mediators of inflammation. Specifically, they attenuate the expression of hepatic Toll‐Like Receptor (TLR) subtypes (TLR2, TLR4, TLR9), the CD14 molecule (which binds endotoxin), and downstream inflammatory pathways. This includes reducing the nuclear abundance of NF‐κB (p50 subunit) and subsequently lowering the expression of inflammatory targets like chemokines (MCP1), cytokines (IL‐1β), inflammasome components (NLRP3), and oxidative stress markers (NOX2 and its subunits). We have previously reported that microalgae oil can efficiently inhibit the expression of genes associated with lipogenesis and promote the expression of genes associated with lipolysis (Ran et al. 2022). Our study revealed that microalgae oil might modulate key pathways involved in lipid and glucose metabolism. It showed potential enhancement of lipid oxidation through PPAR activation while suppression of glucose‐related signaling pathways, such as PI3K‐Akt. These effects align with previous reports on the benefits of omega‐3 PUFAs (Xu et al. 2002), including DHA and EPA, in regulating lipid metabolism and reducing inflammation. *Irs1*, a key gene in insulin signaling, was activated upon microalgae oil treatment enhancing glucose uptake, glycogen synthesis, and lipid metabolism. The reduced expression of pro‐inflammatory genes, such as *Saa2*, and the partial normalization of *Hmgcr* levels in the microalgae oil group might suggest anti‐inflammatory and cholesterol‐suppressing potentials. Furthermore, our metabolomic analysis demonstrated that microalgae oil modulated liver metabolites, including bile acids and fatty acids while partially mitigating the HFD‐induced reduction in amino acids and short‐chain fatty acids (SCFAs). These changes might partially contribute to the overall improvement of lipid and glucose metabolism observed in our study. Hepatic steatosis is closely linked to mitochondrial dysfunction and oxidative stress, both of which are key contributors to NAFLD progression. Studies showed that co‐administration of EPA and the antioxidant hydroxytyrosol significantly mitigated HFD‐induced liver damage. This proved to reduce steatosis severity alongside improvements in mitochondrial function and metabolic regulation, particularly within the AMP‐activated protein kinase (AMPK)–peroxisome proliferator‐activated receptor gamma coactivator‐1α (PGC‐1α) signaling axis. HFD feeding suppressed the expression of AMPK–PGC‐1α components, nuclear respiratory factor‐2 (NRF‐2), and β‐ATP synthase, whereas combined EPA and hydroxytyrosol supplementation markedly reversed these alterations (Echeverría et al. 2019). Together, these findings emphasized the importance of omega‐3–based nutritional strategies in modulating lipid metabolism, oxidative stress, and mitochondrial signaling pathways in NAFLD. In this context, our results using DHA‐rich microalgae oil might further support the therapeutic potential of targeted omega‐3 PUFA interventions in alleviating HFD‐induced hepatic steatosis. DHA functions not only as a long‐chain omega‐3 PUFAs but also as a precursor for specialized pro‐resolving lipid mediators (SPMs), such as resolvins, protectins, and maresins. These bioactive mediators are formed enzymatically from DHA and are increasingly recognized for their anti‐inflammatory and pro‐resolution properties, helping to reduce and actively resolve inflammation across various tissues (Beyer et al. 2023). Unlike traditional pro‐inflammatory eicosanoids, DHA‐derived SPMs influence multiple biological pathways to modulate inflammation and promote tissue repair. In liver disease, DHA‐derived mediators have been associated with hepatoprotective effects by dampening inflammation and facilitating tissue recovery. While clinical data remain limited, studies on omega‐3 PUFAs indicate that DHA may be more effective than EPA in lowering systemic inflammation and markers of hepatic fibrosis. Higher levels of DHA in the liver and circulation were linked with improved anti‐inflammatory profiles and decreased biomarkers of liver injury in NAFLD and NASH models, as well as in some human studies, suggesting enhanced regulation of inflammatory signaling and fibrogenesis when DHA is predominant. Mechanistically, DHA and its lipid mediators interact with immune‐regulating receptors and signaling pathways, suppress pro‐inflammatory cytokine production, and promote a shift toward pro‐resolving macrophage phenotypes. This pro‐resolving activity complements DHA's effects on hepatic lipid metabolism, including inhibition of lipogenesis and stimulation of fatty acid oxidation, collectively supporting better hepatic homeostasis in steatotic livers (Beyer et al. 2023). Although both EPA and DHA positively influence hepatic lipid and inflammatory pathways, DHA appears particularly effective at generating pro‐resolving lipid mediators that counter chronic inflammation and aid liver tissue recovery, which may explain its relatively stronger hepatoprotective effects in certain contexts.

Several limitations to this study should be considered. The relatively small sample size may limit statistical power and the generalizability of the findings. In addition, while the HFD‐induced NAFLD mouse model captures key features of the disease, it does not fully capture the complexity and heterogeneity of NAFLD in humans. The dosing regimen, involving a single dose administered via oral gavage, may not directly translate to typical human intake patterns, where intake occurs through diet and may influence absorption and metabolic responses. Furthermore, dietary intake was not quantitatively evaluated, which may influence metabolic outcomes. While beneficial effects were observed, the underlying molecular mechanisms require further investigation. Despite promising results from several human studies supporting the use of supplemental omega‐3 PUFAs to treat MASLD (Argo et al. 2015; Sanyal et al. 2014), limitations exist, particularly in treating the more severe form, NASH (Argo et al. 2015; Sanyal et al. 2014). For instance, a double‐blind, placebo‐controlled trial found that MASLD patients treated with Lovaza (4 g/d of EPA and DHA‐ethyl esters) for 15 to 18 months achieved a significant reduction in liver fat but failed to achieve a significant reduction in fibrosis scores compared to the placebo group (Scorletti et al. 2014). DHA‐rich microalgae oil has potential as a natural therapeutic for MASLD due to its characteristics in improving lipid and glucose metabolism, reducing liver fat, and modulating key metabolic pathways. While statins (cholesterol biosynthesis inhibitors) (Pockros et al. 2019), and other emerging drugs such as firsocostat (ACC inhibitor) (Matsumoto et al. 2020) and lobeglitazone (PPAR inhibitor) (Lee et al. 2017) show promise in clinical trials, natural products like DHA‐rich microalgae oil offer a safer alternative with multifaceted benefits, including improvements in lipid metabolism, glucose homeostasis, and hepatic function. Future research should focus on optimizing its dosage, exploring its synergistic effects with other therapies, and developing innovative therapies to improve outcomes of MASLD patients.

## Author Contributions

**Liyuan Ran:** methodology. **Chao Liu:** methodology. **Jinhui Yu:** conceptualization, project administration. **Yingjie Wu:** conceptualization, funding acquisition, writing – review and editing. **Mingjie Wang:** investigation, formal analysis, validation. **Liping Liu:** conceptualization. **Yun Liang:** validation. **Qingbo Guan:** supervision. **Athba AlQahtani:** writing – original draft, visualization, formal analysis.

## Funding

This research was funded by the Modern Agricultural Technology Industry System of Shandong Province, China (Grant number: SDAIT‐26) and the Ministry of Science and Technology (“National Key R&D Program of China” No. 2021YFA0805100, and No. 2022YFE0132200).

## Consent

The authors have nothing to report.

## Conflicts of Interest

The authors declare no conflicts of interest.

## Data Availability

All data generated or analysed during this study are included in this published article.
